# Identification and clinical validation of diverse cell-death patterns-associated prognostic features among low-grade gliomas

**DOI:** 10.1038/s41598-024-62869-4

**Published:** 2024-05-24

**Authors:** Wenyong Yang, Hui Yu, Qingqiang Lei, Chunlan Pu, Yuanbiao Guo, Liangbin Lin

**Affiliations:** 1 Medical Research Center, Department of Neurosurgery, Department of Urology, Department of General Surgery, The Third People’s Hospital of Chengdu, The Affiliated Hospital of Southwest Jiaotong University, The Second Chengdu Hospital Affiliated to Chongqing Medical University, Chengdu, 610014 China; 2 Center of Bone Metabolism and Repair, Department of Wound Repair and Rehabilitation Medicine, State Key Laboratory of Trauma, Burns and Combined Injury, Trauma Center, Research Institute of Surgery, Daping Hospital, Army Medical University, Chongqing, 400000 China; 3 Obesity and Metabolism Medicine-Engineering Integration Laboratory, Department of General Surgery, The Third People’s Hospital of Chengdu, The Affiliated Hospital of Southwest Jiaotong University, Chengdu, 610031 China; 4 The Center of Gastrointestinal and Minimally Invasive Surgery, Department of General Surgery, The Third People’s Hospital of Chengdu, The Affiliated Hospital of Southwest Jiaotong University, Chengdu, 610031 China

**Keywords:** Diverse programmed cell death, LGG, Tumor immunology, Prognostic features, *CLU*, *FHL3*, *GIMAP2*, *HVCN1*, Cancer, Neuroscience, Biomarkers

## Abstract

Low-grade glioma (LGG) is heterogeneous at biological and transcriptomic levels, and it is still controversial for the definition and typing of LGG. Therefore, there is an urgent need for specific and practical molecular signatures for accurate diagnosis, individualized therapy, and prognostic evaluation of LGG. Cell death is essential for maintaining homeostasis, developing and preventing hyperproliferative malignancies. Based on diverse programmed cell death (PCD) related genes and prognostic characteristics of LGG, this study constructed a model to explore the mechanism and treatment strategies for LGG cell metastasis and invasion. We screened 1161 genes associated with PCD and divided 512 LGG samples into C1 and C2 subtypes by consistent cluster analysis. We analyzed the two subtypes' differentially expressed genes (DEGs) and performed functional enrichment analysis. Using R packages such as ESTIMATE, CIBERSOTR, and MCPcounter, we assessed immune cell scores for both subtypes. Compared with C1, the C2 subtype has a poor prognosis and a higher immune score, and patients in the C2 subtype are more strongly associated with tumor progression. LASSO and COX regression analysis screened four characteristic genes (*CLU*, *FHL3*, *GIMAP2*, and *HVCN1*). Using data sets from different platforms to validate the four-gene feature, we found that the expression and prognostic correlation of the four-gene feature had a high degree of stability, showing stable predictive effects. Besides, we found downregulation of CLU, FHL3, and GIMAP2 significantly impairs the growth, migration, and invasive potential of LGG cells. Take together, the four-gene feature constructed based on PCD-related genes provides valuable information for further study of the pathogenesis and clinical treatment of LGG.

## Introduction

Glioma is a broad term describing neuroepithelial tumors of glial or supportive cells originating in the central nervous system (CNS)^1^. Glial cells are the most abundant cell type in the CNS, surrounding, isolating, and providing nutrients and oxygen to neurons. WHO classifies gliomas as restricted gliomas (WHO I), low-grade gliomas (LGG; WHO II-III), and malignant gliomas (GBM; WHO IV)^2^. Gliomas constitute 24% of all primary brain and central nervous system tumors. These neoplasms exhibit a wide range of histological characteristics, spanning from benign ependymomas to grade IV glioblastoma multiforme, which is the most aggressive and lethal form^3,4^. Malignant brain tumors are more common in men, while meningiomas and other benign tumors are more common in women. The incidence of glioma varies according to its histological subtype. Pilocytic astrocytomas are more prevalent among children and adolescents, while LGG exhibit a peak incidence between the ages of 30 and 40. GBM, on the other hand, predominantly affects individuals aged 60 to 70 years^5,6^.

LGGs are slow-growing glial tumors with malignant transformation potential. In 2021, WHO reclassified LGG based on classical histopathological features and key molecular markers, including isoacid dehydrogenase (IDH) mutations and 1p/19q co-deletion status, reflecting a stronger correlation between prognosis and molecular diagnostic features^2^. Despite recent advancements in the diagnosis and treatment of LGGs, some patients may experience malignant transformation of their LGGs into high-grade gliomas (HGGs). This progression results in a diminished response to therapeutic interventions and a poorer overall prognosis for the disease^7^. Consequently, there is an urgent need to identify specific and practical molecular signatures that can facilitate accurate diagnosis, enable individualized therapeutic approaches, and provide reliable prognostic evaluations for patients with LGGs^8,9^.

According to the triggering mechanism, cell death is divided into accident cell death (ACD) and programmed cell death (PCD). ACD is an uncontrolled biological process, whereas PCD involves tightly regulated signaling cascades and molecular effector mechanisms. So far, the defined PCD includes apoptosis, autophagy-dependent cell death, lysosome-dependent cell death, necroptosis, ferroptosis, cuproptosis, pyroptosis, netotic cell death, entotic cell death, parthanatos, oxeiptosis, and alkaliptosis^10–12^.

Autophagy-dependent cell death is a kind of PCD driven by the molecular mechanism of autophagy. The process of autophagy involves the sequential formation of three distinct membrane structures, namely, the phagosome, the autophagosome, and the autolysosome. More than 40 autophagy-related genes/proteins (ATGs) play key roles in autophagy membrane dynamics and processes^13–16^. Apoptosis is the process by which living organisms remove damaged or unnecessary cells. Apoptotic vesicles are phagocytosed by macrophages, leaving surrounding cells undisturbed, which does not trigger an inflammatory response^17,18^. Lysosome-dependent cell death (LCD) is a form of PCD mediated by hydrolytic enzymes that are released into the cytosol after lysosomal membrane permeabilization^19^. Necroptosis is a kind of programmed necrosis with morphological characteristics similar to necrosis. Necrosis can be caused by a variety of stimuli, including death receptors (TNFRSF1A and FAS), nucleic acid sensors (Z-DNA–binding protein 1 [ZBP1, also known as DAI], retinoic acid receptor responder 3 [RARRES3, also known as RIG1], transmembrane protein 173 [TMEM173, also known as STING]), toll-like receptors (toll-like receptor 3 [TLR3] and TLR4), and adhesion receptors^20^. Ferroptosis is a type of PCD that depends on iron and lipotoxicity. Ferroptosis can be induced in a typical manner by inactivating GPX4, the main protective mechanism of biofilms against peroxidation damage, and in an atypical manner by increasing the labile iron pool^21,22^. Cuproptosis is a newly discovered PCD triggered by copper, which correlates with multiple diseases^23^. Pyroptosis is a form of PCD driven by the activation of the inflammasome, which is a cytoplasmic multiprotein complex responsible for the release of interleukin (IL) 1 family members (IL1B and IL18), the formation of apoptosis-associated speck-like proteins (ASC) spots, and the activation of pro-inflammatory caspase^24^. Parthanatos is activated by oxidative stress-induced DNA damage and chromatinolysis, which is a form of PCD dependent on poly [ADP-Ribose] polymerase 1 (PARP1)^25^. Oxeiptosis is driven by the activation of the KEAP1-PGAM5-AIFM1 pathway, and it’s a novel oxygen radical-induced caspase-independent PCD^26^. Alkaliptosis is a novel type of RCD driven by intracellular alkalinization^27^.

Cell death can manifest in various forms as a response to different stressors, particularly oxidative stress^28^. A loss of control over single or mixed modes of cell death can contribute to the development of human diseases, such as cancer, neurodegenerative disorders, autoimmune conditions, and infectious diseases^29–32^. Many studies have shown that PCD plays an important role in the development and metastasis of tumors, cells that do not drip PCD properly may develop into malignant tumor cells^33^. However, the precise role of PCD in LGGs has been less extensively studied. Investigating the alterations in PCD pathways in LGGs may provide new markers or therapeutic targets, potentially improving the prognosis and treatment outcomes for patients with these tumors. In addition, the prognostic value of PCD-related genes and the tumor microenvironment (TME) in LGG remain unknown. In this study, we investigated the expression and significance of PCD-related genes in LGG and established a new index, cell death RiskScore (CDR), to predict the efficacy and prognosis of LGG therapeutic interventions.

## Materials and methods

### Data acquisition and preprocessing

The clinical follow-up information and tissue expression data of LGG patients, including astrocytomas and low-grade oligodendrogliomas, were downloaded from the TCGA public database and CGGA database, and the data were processed as follows: (1) The samples without clinical follow-up information were removed. (2) Transform the set into gene symbol. (3) Take the maximum value when multiple Gene Symbol expressions exist. (4) Samples without expression profile data were removed.

PCD-related genes contained 12 key regulatory genes of PCD, including 580 apoptosis-related genes, 367 autophagy-related genes, 220 lysosome-dependent cell death-related genes, 101 necroptosis-related genes, 88 ferroptosis-related genes, 52 pyroptosis-related genes, 15 entotic cell death-related genes, 14 cuproptosis-related genes, 9 parthanatos-related genes, 8 netotic cell death-related genes, 7 alkaliptosis-related genes, and 5 oxeiptosis-related genes. Finally, 1161 PCD-related genes were included in the analysis (Table S1)^11,12^.

After preprocessing, 512 samples were obtained from TCGA-LGG, and 420 patients from CGCA-mRNAseq_693, 172 patients from CGCA-mRNAseq_325, and 159 patients from CGCA-mRNA-array_301 databases, respectively (Table1).

**Table 1:** Cohorts information.

Clinical features	TCGA-LGG	CGCA-mRNA-array_301	CGCA-mRNAseq_325	CGCA-mRNAseq_693
OS				
0	386	85	82	224
1	126	74	30	197
DFS				
0	308			
1	165			
Grade				
G2	248			
G3	263			
IDH mutation				
WT	293			
Mut	127			
Gender				
Male	285			
Female	227			
Age				
≥ 60	69			
< 60	443			
Recurrence				
Yes	165			
No	310			

### Consistent cluster analysis using the ConsensusClusterPlus algorithm

The TCGA expression profile data were filtered to remove genes whose expression level was less than 1, and then univariate COX analysis was performed under the threshold of P < 0.05 to filter out unnecessary genes. ConsensusClusterPlus was used to uniformly cluster TCGA samples by obtaining PCD genes associated with prognosis (V1.48.0; Parameters: pFeature = 1, pItem = 0.8, distance = "spearman ", rep = 100).

### Division of training set and verification set

According to the ratio of training set: verification set = 1:1, 512 samples in the TCGA data set were randomly sampled into groups, which were divided into the training set and verification set. The training set and validation set were selected according to the following conditions: (1) The age distribution, gender, follow-up time, and mortality ratio of patients in the two groups were similar; (2) After the clustering of gene expression profiles, the number of samples was similar between random groups. This selection generates 256 samples in the training set and 256 samples in the verification set.

### Lasso cox regression analysis

To reduce the number of genes found in the risk model, we used lasso regression for prognostic genes. The lasso method results in a finer model by constructing a penalty function that allows the coefficient to shrink, setting part of the coefficient to zero, and preserving the advantage of subset shrinkage. R-package glmnet was used for lasso cox regression to analyze the motion trajectories of each variable. Then, the optimal model is constructed through five cross-validations, and the confidence interval under each lambda is analyzed to find out the number of target genes.

### Cell transfection

Small interfering RNA against negative control (NC-siRNA), CLU (CLU-siRNA), FHL3 (FHL3-siRNA), GIMAP2 (GIMAP2-siRNA) and HVCN1 (HVCN1-siRNA) were all synthesized by GenePharma (Shanghai, China). The sequences were as follows: si CLU-Homo-880, 5′-CCCGCCAACAGAAUUCAUATT-3′; si-CLU -Homo-1206, 5′-GCGAAGACCAGUACUAUCUTT-3′; si FHL3-Homo-179, 5′- GAGCGAGUCAUUUGACUGUTT-3′; si FHL3-Homo-816, 5′- GGAGAACUCUUUGCACCUATT-3′; si GIMAP2 -Homo-617, 5′- GCGAAUCUGUGCCUUUAAUTT-3′; si GIMAP2 -Homo-524, 5′- CCACAAGGAAGACCUCAAUTT-3′; si HVCN1-Homo-519, 5′- CAGCCCGACAAGAAUAACUTT-3′; si HVCN1-Homo-247, 5′- CCUGGAACAUCAACUACAATT-3′; Cell transfection was conducted by using Hieff Trans Liposomal Transfection Reagent (Yeasen Biotechnology (Shanghai)) according to the manufacturer’s protocol.

### MTT and CCK-8 assays

Cell viability assays were conducted on SW1088 cells using two different methods: MTT and CCK-8 assays. SW1088 cells were a gift by Dr. Jingwen Jiang from Sichuan University. SW1088 cells were plated in a 96-well plate at a density of 3000 cells per well and cultured in 100 µl of DMEM supplemented with 10% FBS. For the CCK-8 assay, 10 µl of CCK-8 solution, which was previously diluted in 100 µl of complete culture medium, was added to each well. After incubating the cells for 1 h at 37 °C in the dark, the cell viability was assessed by measuring the absorbance at 450 nm using a microplate reader. In the MTT assay, 10 µl of MTT solution was added to each well after 48 h of cell culture, followed by a 2-h incubation. The culture medium was then removed, and the optical density was measured at 490 nm using a microplate reader.

### Wound healing assay

To perform the wound healing assay, SW1088 cells were seeded in a six-well plate and allowed to grow until a complete monolayer was formed. Subsequently, a controlled scratch was made across the cell layer using a 200-µl plastic pipette tip to create a "wound." The cells were then gently washed three times with PBS to remove any debris. After creating the scratch and washing, we used the standard culture medium without fetal bovine serum to observe cell migration. Time-lapse images of the wounds were captured at specific intervals using an IX71 inverted microscope from Leica Corporation. These images were further analyzed using ImageJ software to measure the rate of cell migration and wound closure. The assay was conducted in triplicate to ensure accuracy and reproducibility, following a predetermined study protocol.

### Transwell assay

The transwell assay is a widely used method to assess cell invasion potential. In this assay, Matrigel-coated transwell chambers are employed to mimic the extracellular matrix and create a barrier for cell migration. Transfected cell lines are cultured and seeded in the upper chamber, which is filled with serum-free medium. The lower chamber is filled with complete culture medium, providing a chemoattractant for cell migration. During the incubation period, cells with invasive properties can penetrate the Matrigel layer and migrate towards the lower chamber. Non-invading cells remaining in the upper chamber are carefully removed. The invaded cells in the lower chamber are then fixed, stained, and visualized under a microscope. Quantification of cell invasion is performed by counting the number of invaded cells in multiple fields of view. Typically, ten randomly selected fields are analyzed to obtain a representative measurement. This allows for a reliable assessment of cell invasiveness.

### Statistical analysis

Data are presented as means ± SEM. Two-tailed unpaired t test was used to compare the difference between two groups. One-way ANOVA followed by Turkey’s multiple comparisons test was used to compare differences between multiple groups. An adjusted P‐value < 0.05 was considered statistically significant. The level of significance is indicated as *, P < 0.05; **, P < 0.01; ***, P < 0.001. Statistical analysis was performed using Prism software.

## Results

### Workflow of this study

**Figure 1:**
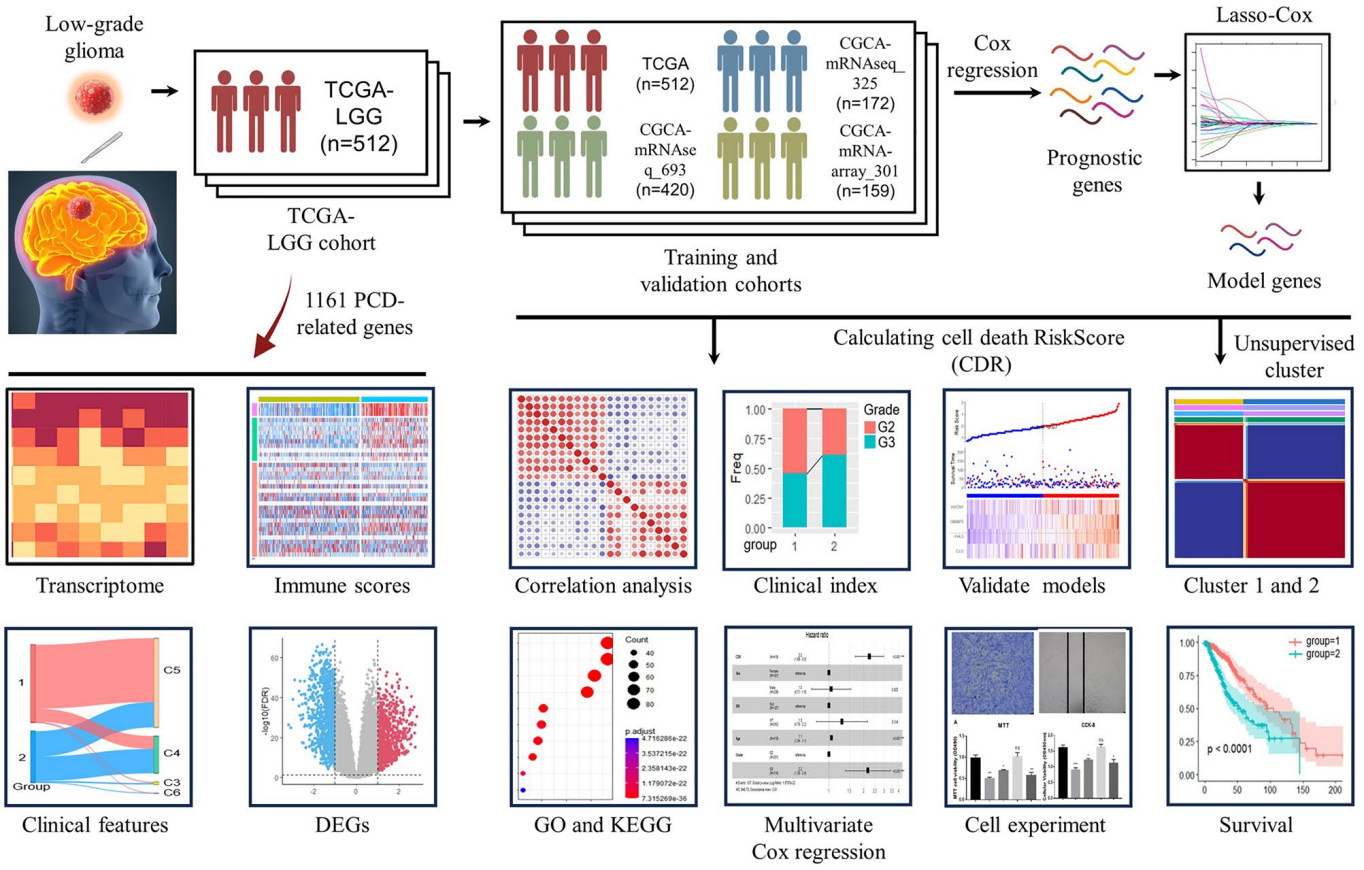
Flowchart for comprehensive analysis of programmed cell death in postoperative patients with LGGs.

We identified 512 patients from TCGA-LGG, 420 patients from CGCA-mRNAseq_693, 172 patients from CGCA-mRNAseq_325, and 159 patients from CGCA-mRNA-array_301 databases for training and validation cohorts. And, 1161 PCD-related genes were brought into the analysis. The flow diagram of this study is showed in the Fig. 1.

### Identification of subtypes based on PCD-related gene

**Figure 2:**
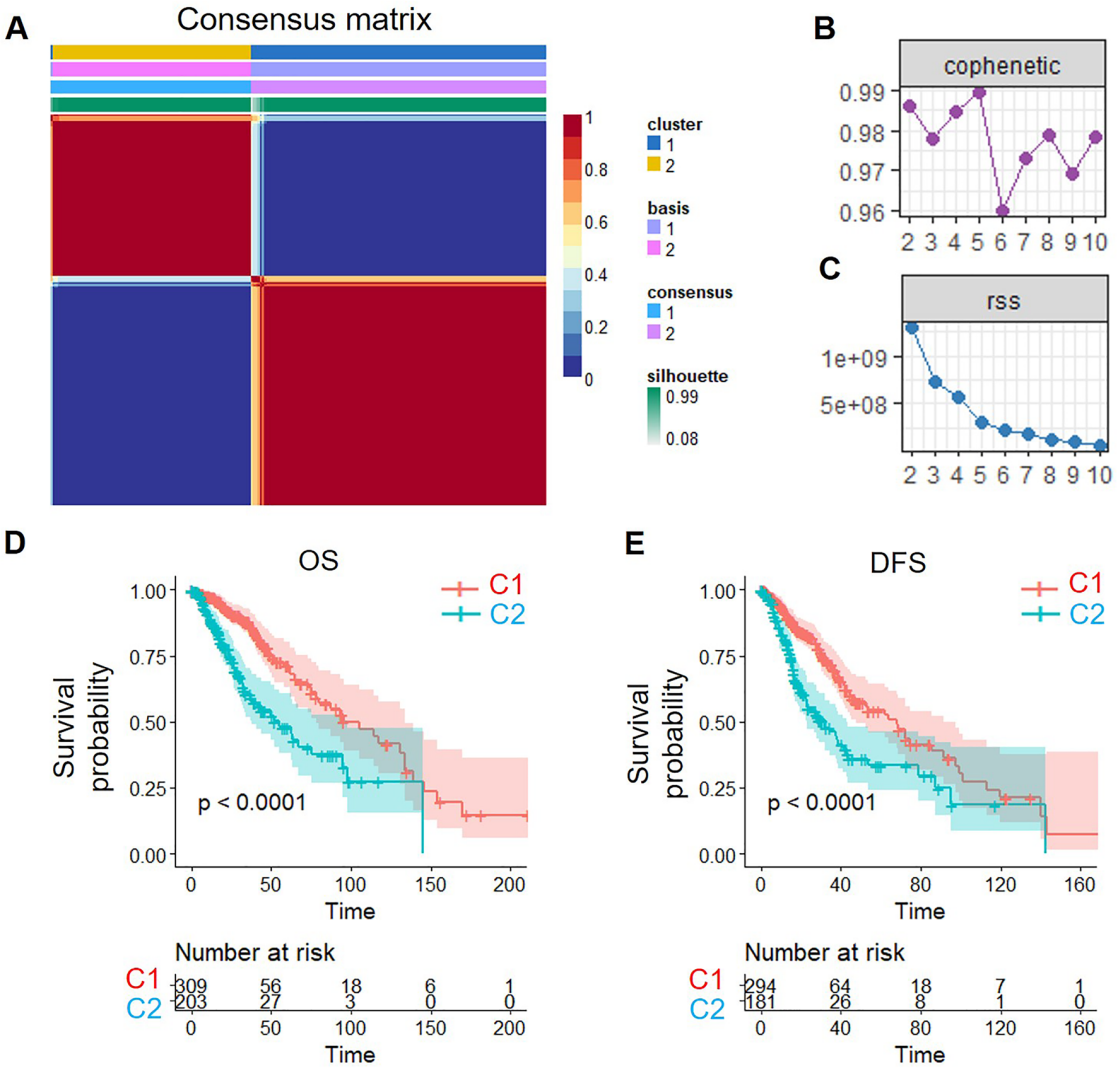
Characterizing LGG subtypes: NMF clustering, prognostic survival, and disease progression. (**A**) Consensus map of NMF clustering. (**B**,**C**) The cophenetic distribution (**B**) and RSS distribution (**C**) at rank = 2–10. (**D**,**E**) Overall survival (OS) time prognostic survival curve (**D**) and disease-free survival (DFS) prognostic survival curve (**E**) of LGG subtypes.

We extracted the expression of 1161 PCD-related genes from the TCGA LGG dataset. 590 genes associated with LGG prognosis were identified by univariate cox analysis in R (Table S2) (*P* < 0.05). Non-negative matrix factorization (NMF) was performed to cluster LGG samples based on the expression of 590 genes. The optimal clustering of k = 2 was selected by synthesizing and residuals sum of squares, and two subtypes (C1 and C2) were obtained (Fig. 2A–C).

Subsequently, we conducted a comparative analysis of the prognoses associated with the two identified subtypes. Our findings revealed that subtype C2 exhibited a poorer prognosis in terms of both overall survival (OS) (Fig. 2D) and disease-free survival (DFS) (Fig. 2E). These results indicate the existence of significant differences in survival rates between the two subtypes.

### Comparison and analysis of clinical features between subtypes

**Figure 3:**
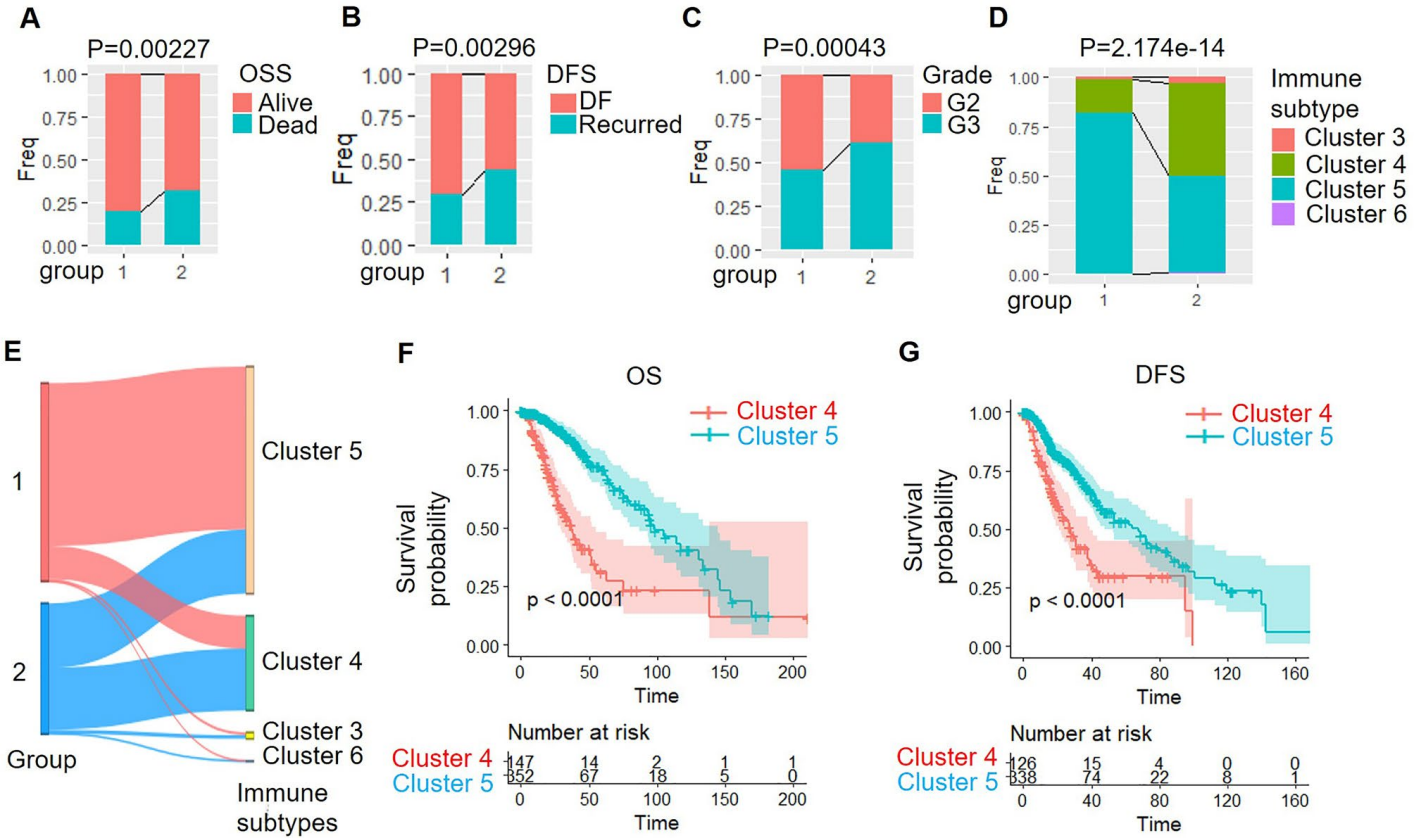
Exploring clinical and immune features of LGG subtypes: survival, tumor characteristics, and immune subtype associations. (**A**–**D**) Comparison of the distribution of two subtypes in clinical features such as survival rate (**A**), tumor recurrence rate (**B**), tumor grade (**C**), and immune subtypes (**D**) in the TCGA data set. (**E**) Comparison of distribution of immune subtypes in the two subtypes. (**F**) Comparison of two subtypes with existing immune subtypes. (**F**–**G**) OS time prognostic survival curve (**F**) and DFS prognostic survival curve (**G**) of LGG subtypes between C4 and C5 immune subtypes.

Next, we analyzed and compared the clinical characteristics of the two subtypes. Our analysis demonstrated that the C1 group exhibited a significantly higher survival rate compared to the C2 group (Fig. 3A), and the tumor recurrence rate within the C1 group was lower than that observed in the C2 group (Fig. 3B). Furthermore, the proportion of cases classified as G3, which is associated with a poor prognosis, was lower in the C1 group relative to the C2 group (Fig. 3C). These results indicated that the C1 group has a better prognosis and clinical features.

There are identified six types of immune infiltration in human tumors, including Cluster 1 (wound healing), Cluster 2 (INF-γ dominant), Cluster 3 (inflammatory), Cluster 4 (lymphocyte depleted), Cluster 5 (immunologically quiet), and Cluster 6(TGF-β dominant). Comparing our stratified method with this stratified method, we found that most LGG are C4 and C5, and the proportion of Cluster 5 in the C1 group was higher than the C2 group (Fig. 3D and E). A further comparative analysis of the prognoses between Cluster 4 and Cluster 5 revealed significant differences in overall survival (OS) (Fig. 3F) and disease-free survival (DFS) (Fig. 3G). Cluster 5 exhibited a superior prognosis compared to Cluster 4, indicating that the PCD-subtype C1 encompasses a higher proportion of immune subtypes associated with favorable prognoses.

### Subtypes with good prognosis had lower immune scores

**Figure 4:**
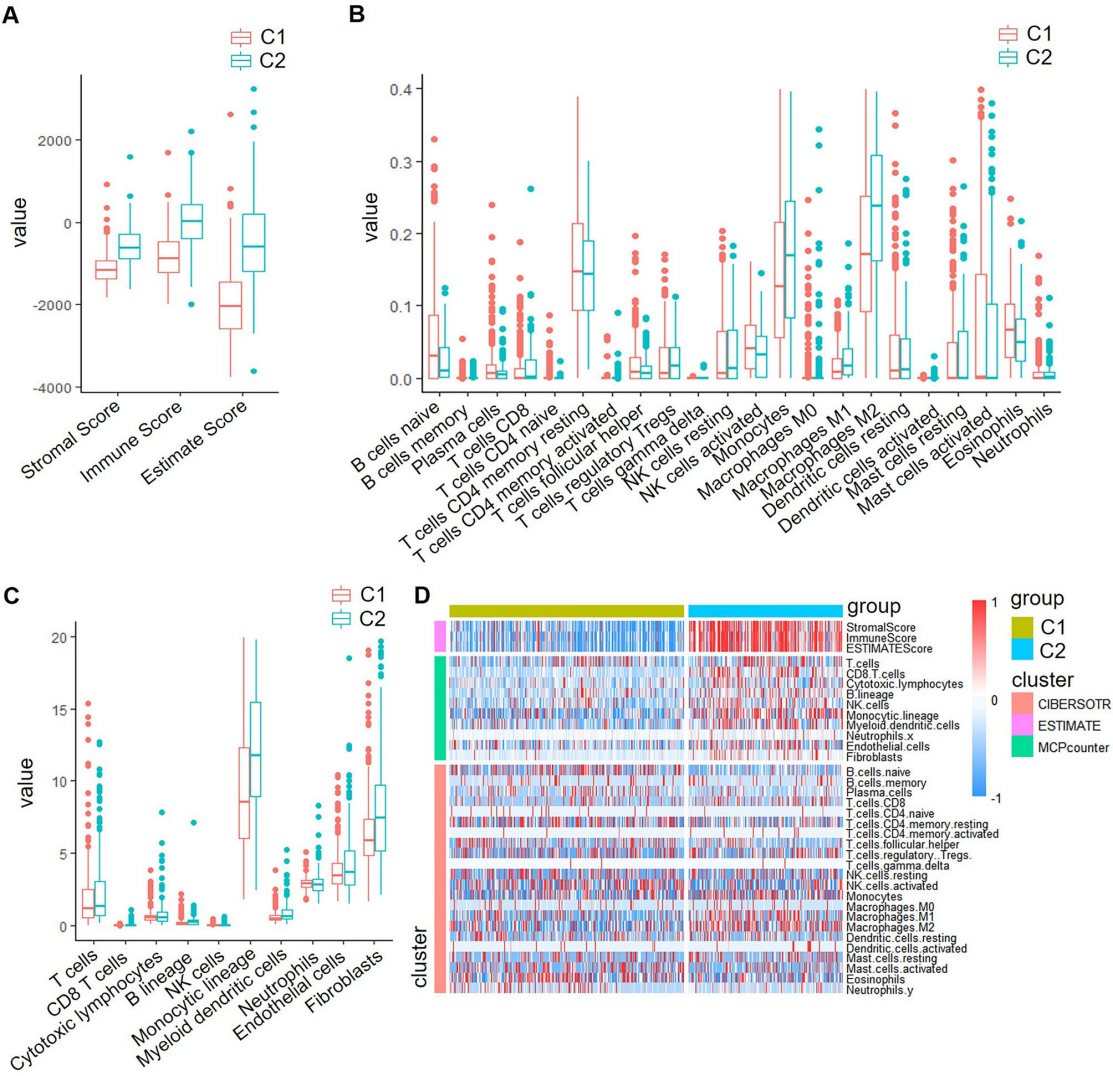
Comparing immune scores and profiles among LGG subtypes: insights from TCGA dataset. (**A**–**C**) Comparison of ESTIMATED immunity scores (**A**), CIBERSOTR immunity scores (**B**), and MCPcounter immune scores (**C**) between subtypes in the TCGA dataset. (**D**) Heat map comparing immune scores among subtypes in the TCGA data set by three immune software.

ESTIMATE package was performed in R software to evaluate three immune scores of the two subtypes (C1 and C2), including the StromalScore, ImmuneScore, and ESTIMATEScore. The results indicated that the C1 subtype exhibited a lower immune score compared to the C2 subtype (Fig. 4A). Subsequently, the MCPcounter and CIBERSORT packages were employed to analyze the scores of ten and twenty-two immune cell types, respectively, between the C1 and C2 subtypes. Our findings revealed that C1 had lower immune cell scores than C2, including T cells, CD8 T cells, B lineage cells, monocytic lineage cells, myeloid dendritic cells, endothelial cells, fibroblasts, M1 macrophages, and M2 macrophages (Fig. 4B and C). Furthermore, the heatmap of immune scores also demonstrated that C1 had lower immune cell scores compared to C2 (Fig. 4D).

### Functional analysis of pathways between subtypes

**Figure 5:**
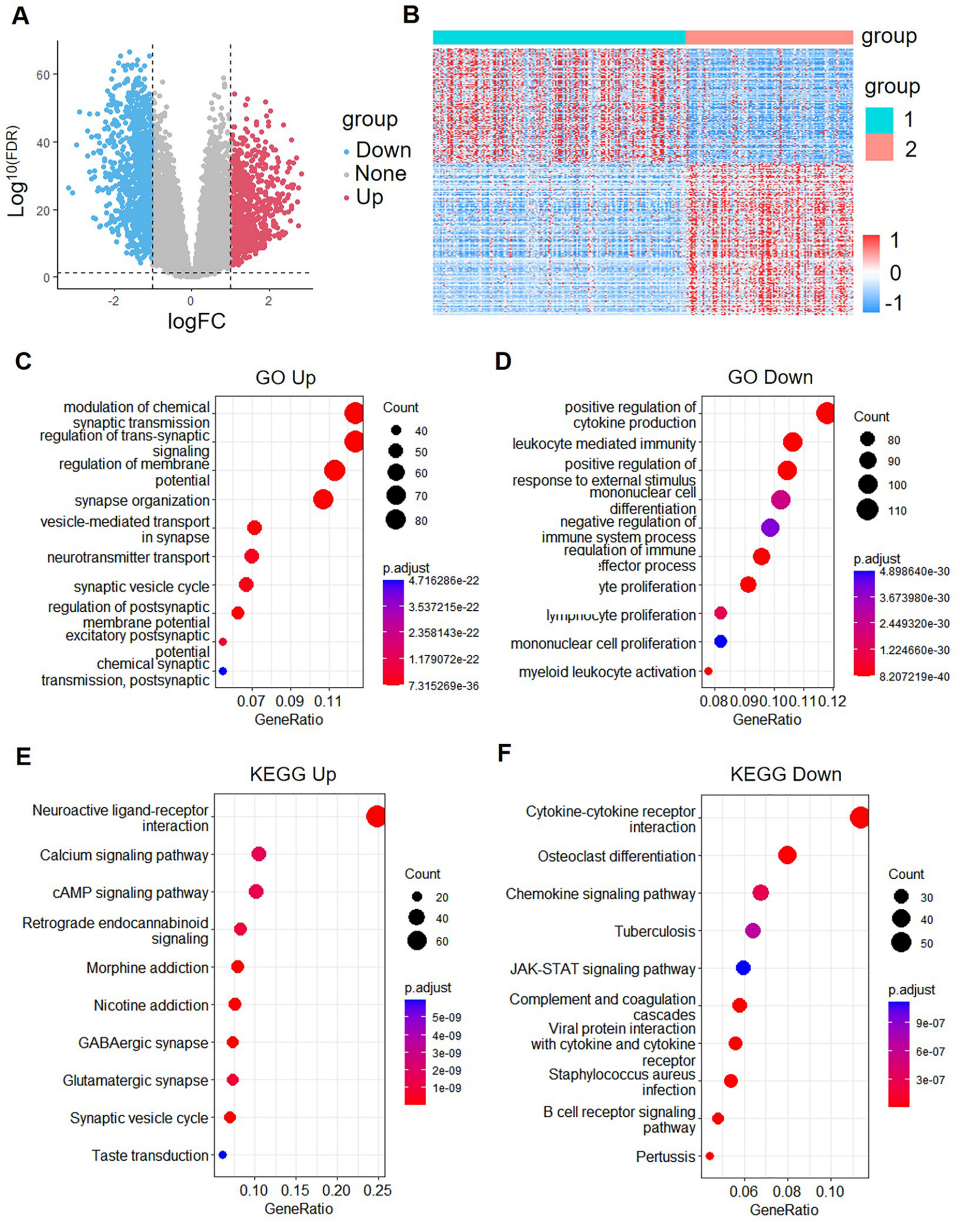
Exploring differentially expressed genes and pathways in LGG subtypes: volcano maps, heatmaps, and functional annotations. (**A**) Volcanic maps of differentially expressed genes (DEGs) of two subtypes. (**B**) Heat maps of DEGs of two subtypes created by the R programming language and the RStudio integrated development environment. (**C**,**D**) Annotated map of biological processes (BP) of subtypes of differentially up-regulated genes (**C**) and subtypes of differentially down-regulated genes (**D**). (**E**,**F**) Annotated KEGG map of subtypes differential up-regulation gene (**E**) and subtypes differential down-regulation gene (**F**).

To identify the DEGs of two subtypes, we calculated 2372 DEGs, containing 1430 up-regulated genes and 942 down-regulated genes, by using limma packets and filtering according to the threshold of |log2FC| > 1 and FDR < 0.01 (Fig. 5A). DEGs are shown in Table S3. The heat map showed all the DEGs of C1 and C2 (Fig. 5B).

GO functional enrichment analysis of 1800 DEGs was performed using Goplot R package. The results showed that the main up-regulated pathways enriched were modulation of chemical synaptic transmission, regulation of trans-synaptic signaling, regulation of membrane potential, synapse organization, and vesicle-mediated transport in the synapse (Fig. 5C). Furthermore, the main down-regulated pathways enriched were positive regulation of cytokine production, leukocyte-mediated immunity, positive regulation of response to external stimulus, mononuclear cell differentiation, and negative regulation of immune system process (Fig. 5D).

We also performed KEGG pathway enrichment analysis for the DEGs. We found that the main up-regulated pathways enriched were neuroactive ligand-receptor interaction, calcium signaling pathway, cAMP signaling pathway, retrograde endo cannabinoid signaling, and morphine addiction (Fig. 5E). Moreover, the main down-regulated pathways enriched were cytokine-cytokine receptor interaction, osteoclast differentiation, chemokine signaling pathway, tuberculosis, and JAK-STAT signaling pathway (Fig. 5F).

### Construction of the prognostic risk model

To build the RiskScore model, 512 patients were randomly grouped, and the training set and the test set both had 256 samples. 590 prognosis-associated genes were identified by using univariate regression Cox risk model analysis of survival data (P < 0.01 was selected as threshold filtering) (Table S4).

**Figure 6:**
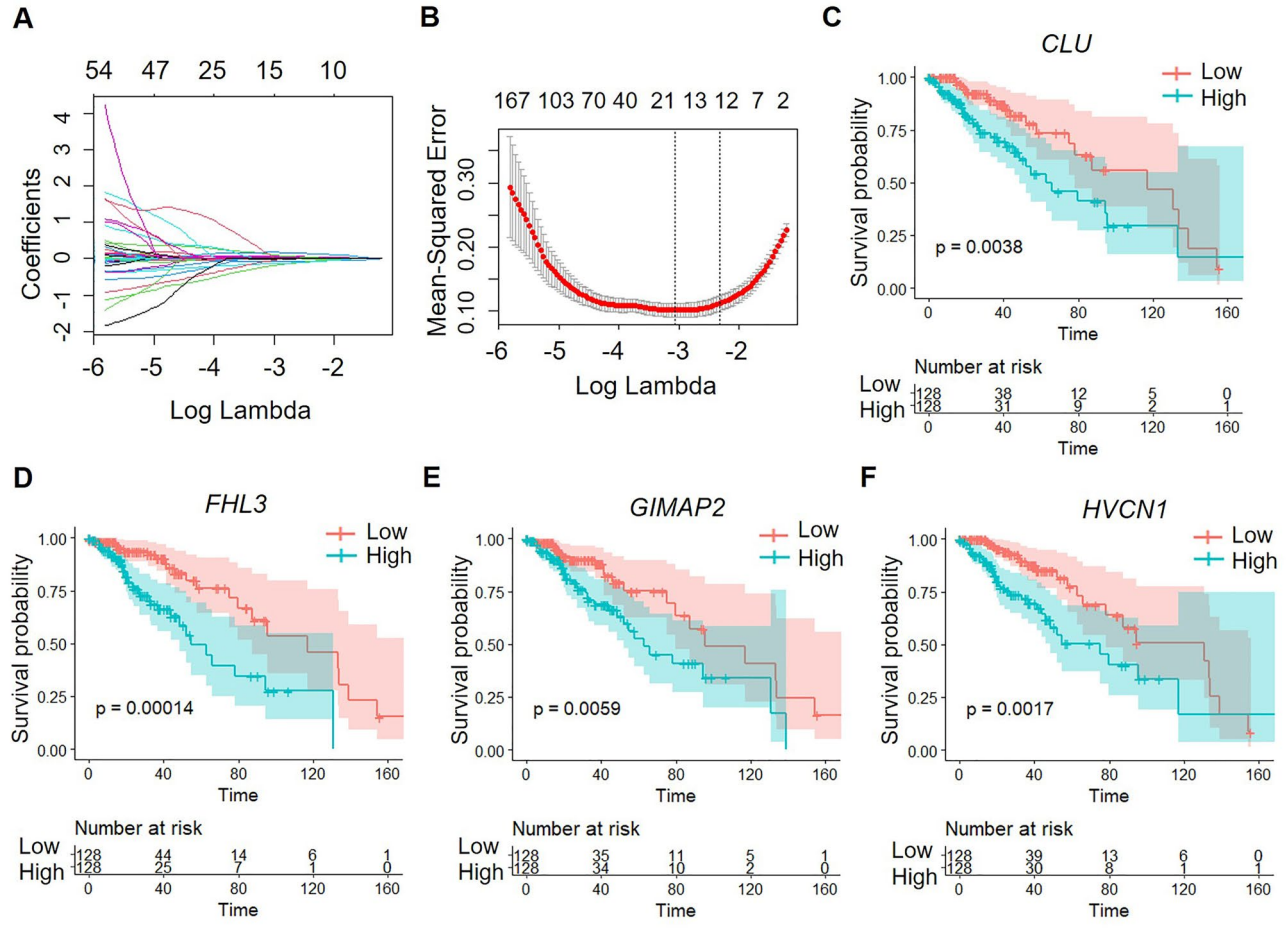
Investigating independent variable trajectories and survival associations of key genes in LGG: coefficients, confidence intervals, and KM curves. (**A**) Independent variable trajectories: vertical axis (representing the coefficient of the independent variable) and horizontal axis (representing the logarithm of the dependent variable). (**B**) Confidence intervals for each λ. C-F. KM Curves of *CLU* (**C**), *FHL3* (**D**), *GIMAP2* (**E**), and *HVCN1* (**F**) in TCGA training set.

To reduce the number of genes, the trajectories of the independent variables were analyzed using lasso Cox regression analysis with the R-package glmnet (Fig. 6A). The confidence intervals under each lambda value were analyzed through cross-validation. We identified 19 genes as the target genes, indicating that the model is applicable, with lambda = 0.04627505 (Fig. 6B).

To further reduce the number of target genes, we performed the Akaike information criterion (AIC) method to produce a better model with fewer parameters. And we screened four genes (*CLU*, *FHL3*, *GIMAP2*, and *HVCN1*) out of 19 genes. The prognostic KM curves indicated that *CLU*, *FHL3*, *GIMAP2* and *HVCN1* were negatively correlated with the survival of patients in the TCGA training set (Fig. 6C–F).

**Figure 7:**
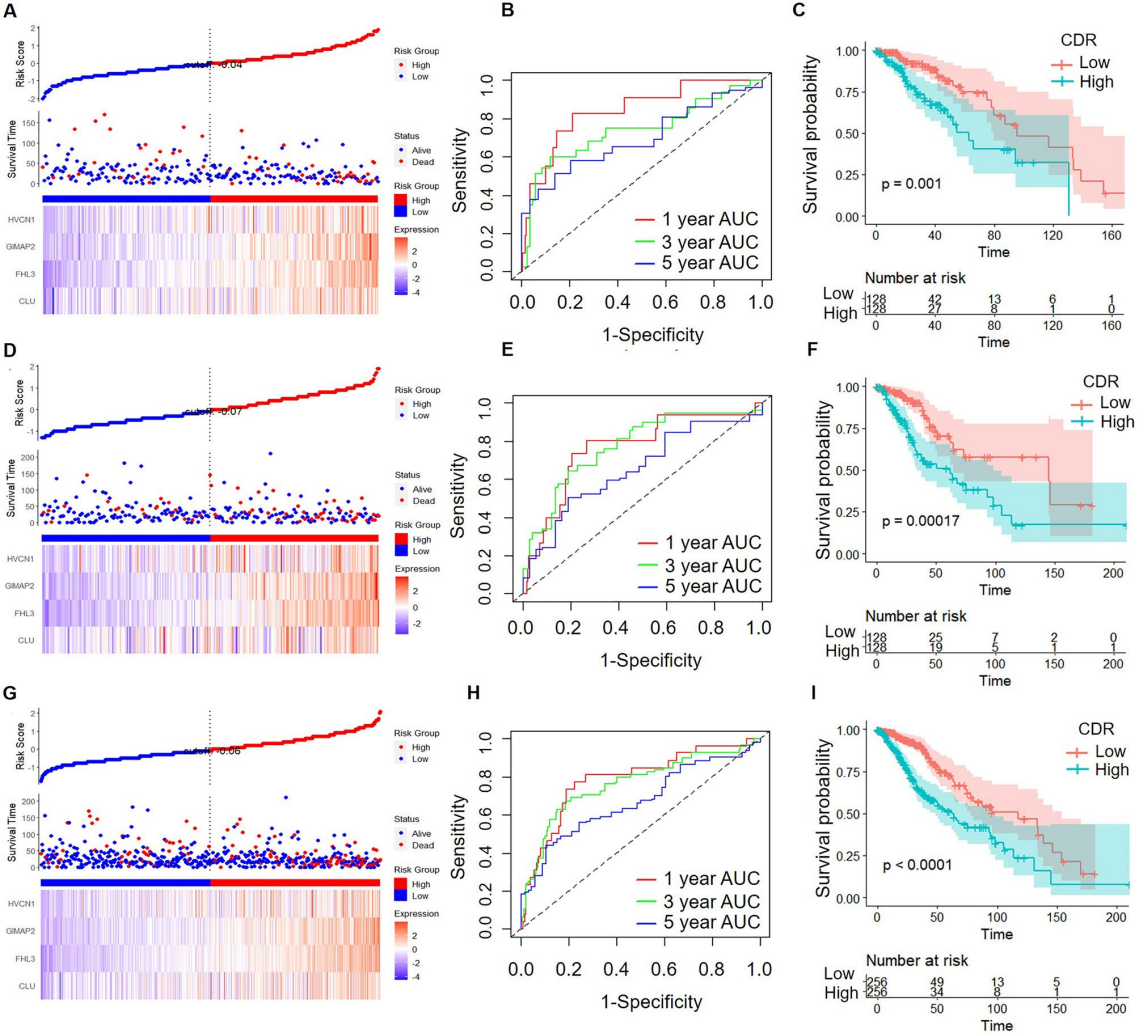
CDR, survival analysis, and classification performance of four-gene features in LGG: TCGA dataset evaluation. CDR, survival time, survival state, and indicated gene expression of the TCGA training set (**A**), the TCGA validation set (**D**), and the full TCGA dataset (**G**). ROC curve and AUCs of four-gene features classification in the TCGA training set (**B**), the TCGA validation set (**E**), and the full TCGA dataset (**H**). The KM survival curve distribution of four-gene features in the TCGA training set (**C**), the TCGA validation set (**F**), and the full TCGA dataset (**I**).

Using the ggRISK package, we calculated a cell death related RiskScore (CDR) for each sample based on the four gene expression levels. The results showed that higher CDR is related to a worse prognosis (Fig. 7A). And using R package timeROC, we analyzed the prognostic prediction efficiency at 1 year, 3 years, and 5 years, and the result showed that the model's Area Under Curves (AUCs) was higher than 0.7 (Fig. 7B). The KM curve showed that the high CDR group had a significantly worse prognosis than the low CDR group (Fig. 7C).

### Validation of the prognostic risk model

To verify the stability of the prognostic risk model, we carried out the same model analysis on the TCGA test dataset. The results showed that higher CDR is related to worse prognosis in the TCGA test dataset (Fig. 7D). And we analyzed the prognostic prediction efficiency at 1 year, 3 years, and 5 years, and we found that the model's AUCs was higher than 0.7 (Fig. 7E). The KM curve showed that the high CDR group had a significantly worse prognosis than the low CDR group in the TCGA test dataset (Fig. 7F).

We further analyzed the distribution of the CDR across the complete TCGA dataset, and the results showed that LGG patients with higher CDR exhibited a worse prognosis (Fig. 7G). The analysis of the prognostic prediction efficiency indicated that the AUC values of the model were higher than 0.7 (Fig. 7H). The Kaplan–Meier curve demonstrated that the high CDR group had a significantly poorer prognosis among all LGG patients (Fig. 7I).

### External datasets verified the prognostic risk model

To further verify the universality and stability of the four gene signatures we screened, three CGGA datasets (CGCA-mRNAseq_301, CGCA-mRNAseq_325, and CGCA-mRNA-array_693 database) were selected to verify the CDR.

**Figure 8:**
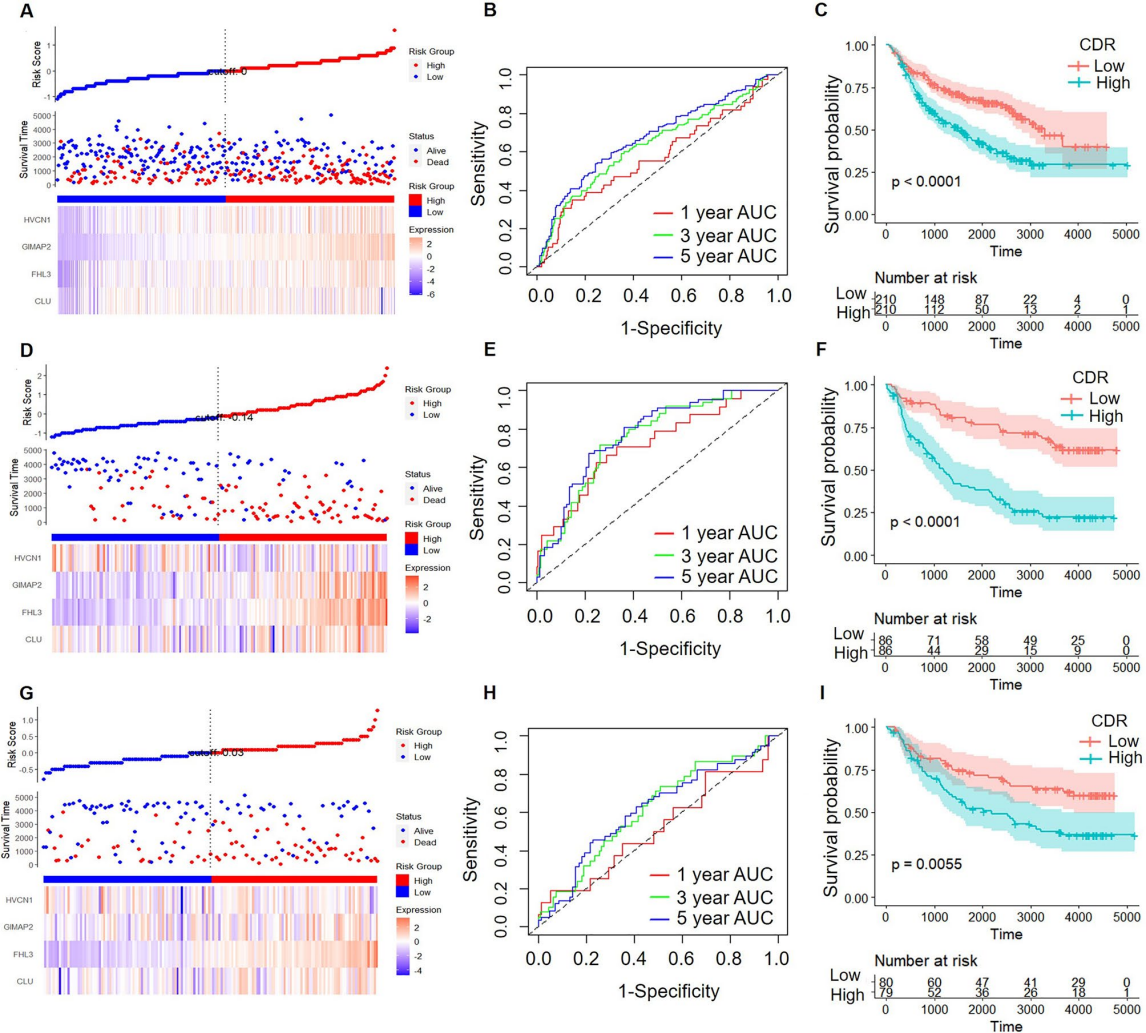
CDR, survival analysis, and classification performance of four-gene features in cgga databases: mRNA expression and prognostic assessment. CDR, survival time, survival state, and indicated gene expression of the CGGA-mRNA-array_301 database (**A**), the CGGA- mRNAseq_325 database (**D**), and the CGGA- mRNAseq_693 database (**G**). ROC curve and AUCs of four-gene features classification in the CGGA-mRNA-array_301 database (**B**), the CGGA- mRNAseq_325 database (**E**), and the CGGA- mRNAseq_693 database (**H**). The KM survival curve distribution of four-gene features in the CGGA-mRNA-array_301 database (**C**), the CGGA- mRNAseq_325 database (**F**), and the CGGA- mRNAseq_693 database (**I**).

Consistent with the above conclusions, higher CDR is related to worse prognosis in all three CGGA datasets (Fig. 8A,D,G). The analysis of the prognostic prediction efficiency indicated that the AUCs of the model were higher than 0.7 (Fig. 8B,E,H). The KM curve showed that the high CDR group had a significantly worse prognosis in all LGG patients (Fig. 8C,F,I).

### Correlation analysis of the prognostic risk model with clinical features and pathways

**Figure 9:**
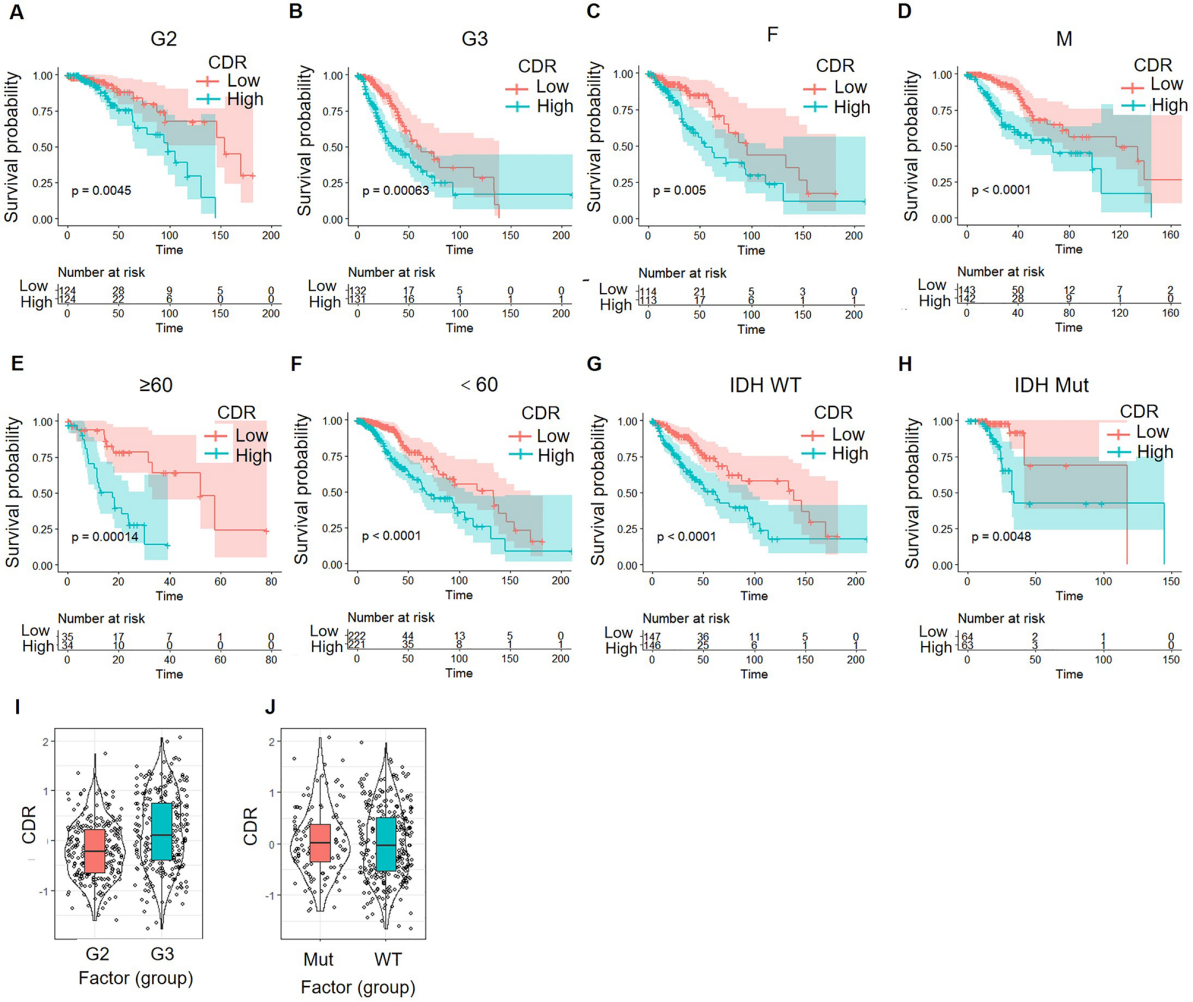
Prognostic significance of CDR in different subgroups of LGG patients: gene characteristics and clinical factors. (**A**–**H**) According to CDR of the four gene characteristics, the patients in G2 group (**A**), G3 group (**B**), female group (**C**), male group (**D**), age ≥ 60 group (**E**), age < 60 group (**F**), IDH WT group (**G**) and IDH mutation group (**H**) were divided into two groups, and the prognosis difference between the two groups was significant. (**I**) CDR in G2 and G3 groups of LGGs. (**J**) CDR in IDH WT group and IDH mutation group of LGGs.

We further analyzed prognostic associations of CDR with different clinical features. In different grade groups, gender groups, age groups, and IDH mutation groups, the group with high CDR had a worse prognosis (Fig. 9A–H), indicating that the model with the four-gene feature had a good predictive ability. Moreover, the higher grade was associated with higher CDR (Fig. 9I,J).

**Figure 10:**
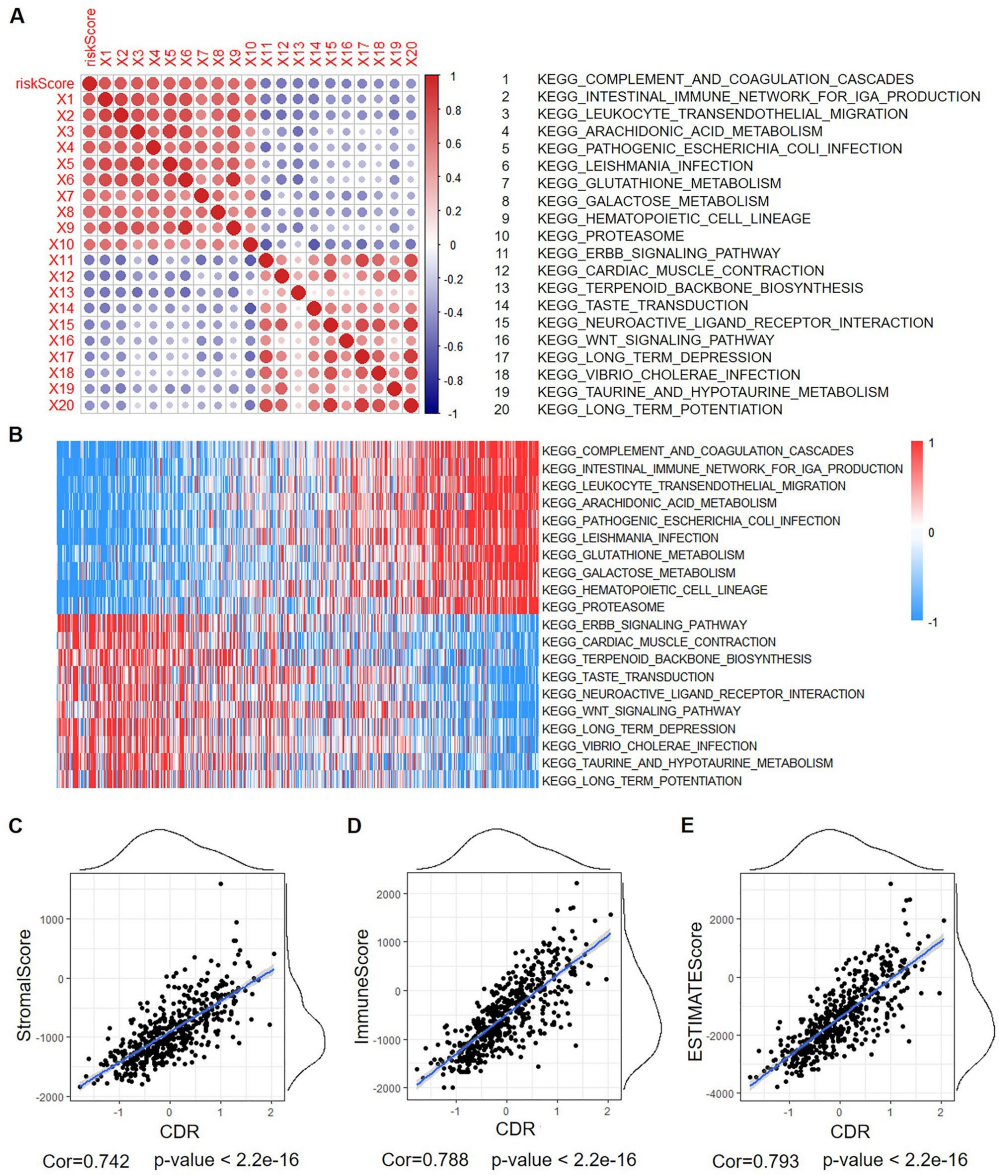
Correlations of CDR with KEGG Pathway Scores, StromalScore, ImmuneScore, and ESTIMATEScore in LGG. (**A**) Correlation between CDR and ssGSEA KEGG signal pathway score. (**B**) Heat map of the KEGG signal pathway. (**C**) The correlation between CDR and StromalScore. (**D**) The correlation between CDR and ImmuneScore. (**E**) The correlation between CDR and ESTIMATEScore.

To study the relationship between CDR and the biological function, we analyzed the gene expression profiles of different samples by single-sample GSEA. We analyzed the correlation between the KEGG pathway and CDR, and we found that the top 10 KEGG positively correlated with CDR, including COMPLEMENT_AND_COAGULATION_CASCADES, INTESTINAL_IMMUNE_NETWORK_FOR_IGA_PRODUCTION, LEUKOCYTE_TRANSENDOTHELIAL_MIGRATION, ARACHIDONIC_ACID_METABOLISM, and PATHOGENIC_ESCHERICHIA_COLI_INFECTION. We also found that the top 10 KEGG pathways that are negatively correlated with CDR, including ERBB_SIGNALING_PATHWAY, CARDIAC_MUSCLE_CONTRACTION, TERPENOID_BACKBONE_BIOSYNTHESIS, TASTE_TRANSDUCTION and NEUROACTIVE_LIGAND_RECEPTOR_INTERACTION (Fig. 10A,B).

We further analyzed the correlation between CDR and immune score and found that the StromalScore, ImmuneScore, and ESTIMATEScore were positively correlated with CDR (Fig. 10C–E).

### Knockdown of CLU, FHL3, and GIMAP2 inhibits growth and migration in LGG cells

**Figure 11:**
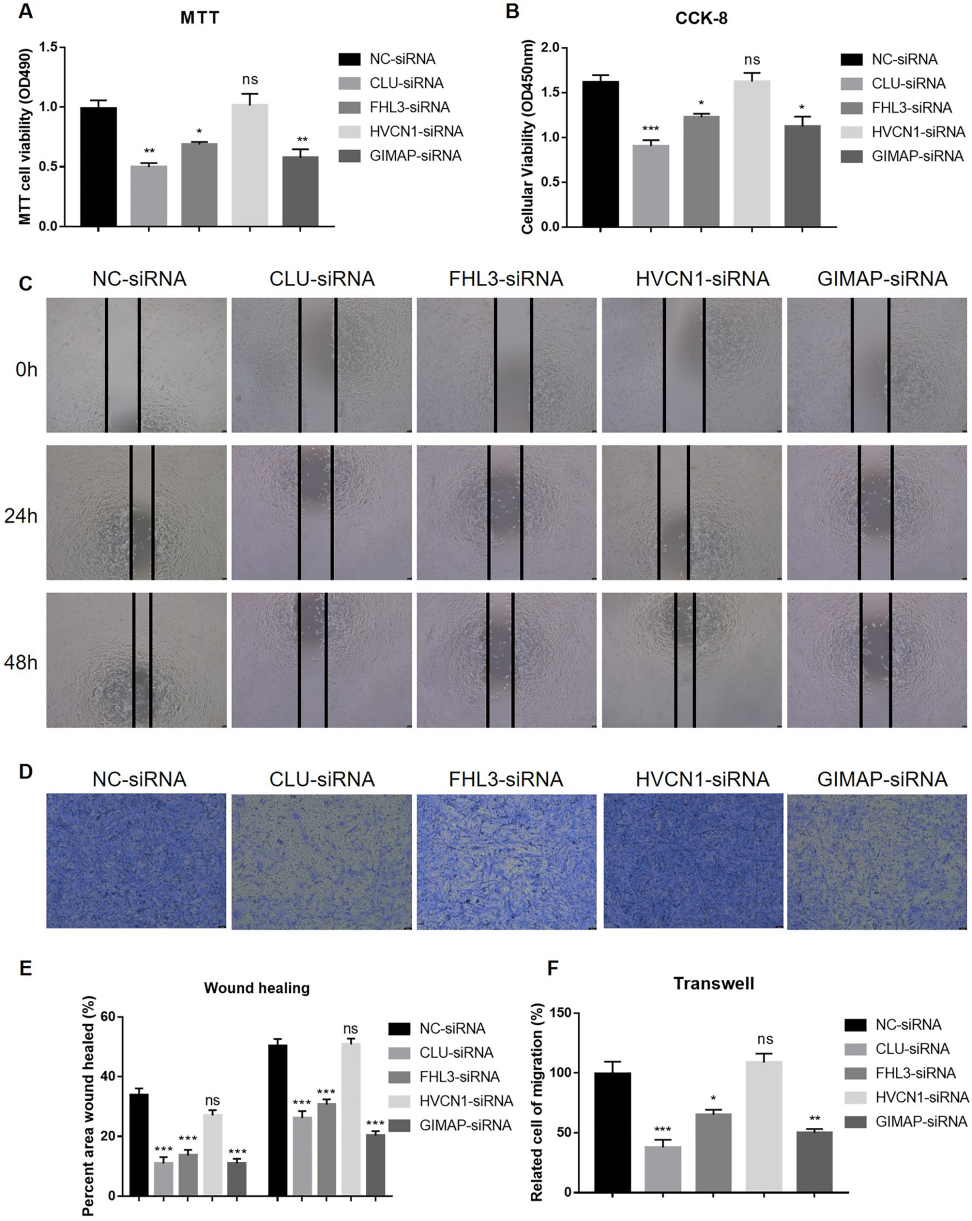
Effects of gene knockdown on cell viability, migration, and invasion in SW1088 Cells: MTT/CCK-8 assays, wound healing, and transwell analysis. (**A**,**B**) Evaluation of cell viability after knockdown of the specified genes using the MTT assay (**A**) and the CCK-8 assay (**B**) in SW1088 cells. (**C**) Examination of cell migratory abilities upon knockdown of the indicated genes using wound healing assays in SW1088 cells. (**D**) Assessment of cell invasive capacities following knockdown of the indicated genes using transwell assays in SW1088 cells. (**E**,**F**) Statistical analysis of the wound healing assays mentioned in C (**E**) and the transwell assays as mentioned in D (**F**).

To further elucidate the roles of four genes in LGG, we individually knocked down these genes in LGG cells (SW1088 cells). Through MTT and CCK-8 experiments, we observed a decrease in cell viability upon knockdown of CLU, FHL3, and GIMAP2, suggesting their roles in suppressing cell growth (Fig. 11A and B). Conversely, knockdown of HVCN1 did not significantly affect cell growth, possibly due to its low mRNA expression levels. Moreover, we conducted wound healing assays, which revealed a weakened migratory ability in cells with CLU, FHL3, and GIMAP2 knockdown (Fig. 11C and E). Consistently, transwell experiments demonstrated a reduced invasive capacity of cells lacking CLU, FHL3, and GIMAP2 (Fig. 11D and F). These findings collectively indicate that CLU, FHL3, and GIMAP2 exert influence on the growth and migration of SW1088 cells. In summary, our results highlight the pivotal roles of CLU, FHL3, and GIMAP2 in modulating the proliferation and migratory capabilities of LGG cells.

**Figure 12:**
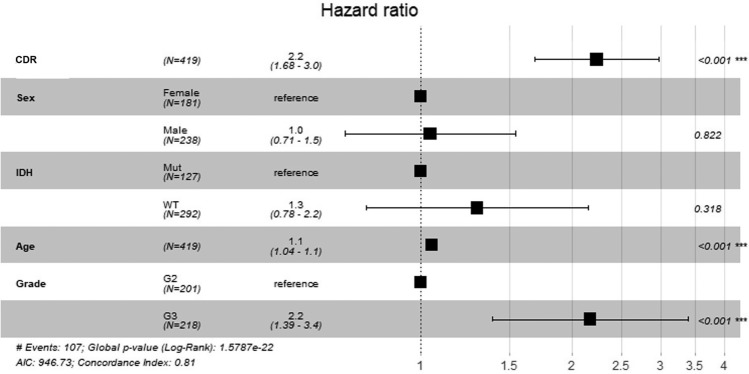
Forest map of multivariate cox regression analysis.

Besides, by multivariate COX regression analysis of the clinical independence of four-gene features in the TCGA dataset, we found that CDR was significantly correlated with survival rate (Fig. 12). Together, these data indicate that our four-gene signature model has good clinical predictive performance.

## Discussion

LGG is a heterogeneous disease regarding molecular characteristics, tumorigenesis, therapeutic response, and clinical outcome. Recurrence or malignant progression of LGG is inevitable even after surgical excision, radiotherapy, chemotherapy, and immunotherapy^8,34^. Consequently, there is an urgent need to identify specific and practical molecular signatures that can facilitate accurate diagnosis, enable individualized therapeutic approaches, and provide reliable prognostic evaluations for patients with low-grade gliomas. Many studies have shown that PCD plays an important role in biological processes and has been confirmed to be related to the occurrence and metastasis of malignant tumors^33,35^. In this study, we constructed a novel four-gene marker (*CLU*, *FHL3*, *GIMAP2*, and *HVCN1*) with good prognostic ability based on PCD-related genes and verified the prognostic ability of this marker in three other independent databases. Based on the expression of the four-gene marker, we established a new index, cell death related RiskScore (CDR), to predict the efficacy and prognosis of LGG therapeutic interventions.

CLU (Clusterin) is a stress-activated, ATP-independent molecular chaperone and is associated with the development of different physiological and pathological processes, including carcinogenesis and tumor progression^36,37^. It plays a crucial role in maintaining protein balance, controlling survival signals, and preventing cell death pathways and genetic regulation^38–40^. Despite limited research on CLU in LGG, our screening results suggest that CLU could serve as a promising prognostic indicator for LGG. Further investigation into its role in LGG is warranted.

FHL3 (four and a half LIM protein 3) play an important role in cardiovascular disease and muscle proliferation by regulating signal transduction and cell growth. FHL3 is often overexpressed or downregulated in different cancers, and there is growing evidence of a link between FHL3 and tumor biology^41^. On the one hand, FHL3 can play a role as a cancer protein in some cancers, promoting tumor progression through phosphorylation. On the other hand, FHL3 can act as a tumor suppressor and affect the expression of downstream genes. Thus, FHL3 is thought to have a dual role in cancer progression, reflecting its complex role in cancer^42^. FHL3 serves as a binding partner of GSK3β and facilitates tumor metastasis in Pancreatic Ductal Adenocarcinoma (PDAC) by impeding the ubiquitin-mediated degradation of snail1 and twist1^43^. In glioma, FHL3 acts as a stemness suppressor in regulating the Smad2/3–SOX4–SOX2 axis^44^. Our screening indicates that FHL3 may serve as a prognostic indicator for LGG, potentially attributed to its role in the Smad2/3–SOX4–SOX2 axis.

GIMAP2 (GTPases of immunity-associated protein 2) belongs to the GIMAPs family, a unique family of GTPases that control lymphocyte apoptosis and play a central role in lymphocyte maturation and lymphocyte-related diseases^45^. Limited research has been conducted on GIMAP2. GIMAP2 was found to form a GTP-dependent scaffold, with its C-terminal amino acid extension guiding it to the lipid droplet. GIMAP2 expression was consistently observed in all examined human lymphoma T cell lines, while other GIMAP members were suppressed in these tumor cell lines^45^. Abnormal GIMAP2 expression affects the progression of oral squamous cell carcinoma by promoting the cell cycle and inhibiting cell apoptosis^46^. As a newly discovered protein, GIMAP2 has been identified through screening as a potential prognostic marker for LGG. Further investigation into its function in LGG is warranted.

HVCN1 (hydrogen voltage-gated channel (1) is the only mammalian voltage-gated proton channel. In human B lymphocytes, HVCN1 binds to the B cell receptor (BCR) and is required to optimize BCR signaling and REDOX control. HVCN1 has expressed in BCR-signaling dependent malignant B cells, such as chronic lymphocytic leukemia (CLL) cells^47^. HVCN1 not only controls signaling following B-cell receptor activation and histamine release in basophils but also plays a role in pH-dependent sperm activation and acid secretion in the tracheal epithelium^48^. While the role of HVCN1 in tumor progression remains largely unexplored, our screening has identified HVCN1 as a potential prognostic marker for LGG.

In our study, we constructed subtypes of LGG models based on PCD-associated genes. Using DEGs identified in LGG subtypes, we constructed a four-gene signature (*CLU*, *FHL3*, *GIMAP2* and *HVCN1*) prognostic risk model and validated it using CGGA gene expression data sets. A risk index (Cell Death related RiskScore, CDR) was computed using the expression levels of four gene markers. Our analysis revealed a strong correlation between the four gene markers, CDR, and the invasion, growth, and metastasis of LGG tumor cells, indicating that the four-gene signature serves as robust biomarkers for predicting the prognosis of LGG.

While our findings have revealed intriguing phenomena and novel gene signatures, they are not without limitations. Although the model we developed using ATGC was effectively validated with gene expression data from the CGGA database, additional data from multiple platforms is required to further confirm the validity of our model. The four genes identified in this study warrant further investigation through cell experiments and animal studies to delineate their role in PCD and LGG, thereby establishing a foundation for their potential clinical application in LGG.

### Supplementary Information

Supplementary Table S1.

Supplementary Table S2.

Supplementary Table S3.

Supplementary Table S4.

## Data Availability

The data from the TCGA and CGGA data sets in this study are publicly available.
